# Surface roughness in microfluidic device fabrication: limitations of conventional methods and a novel solution for multi-material bonding†

**DOI:** 10.1039/d5ra02701b

**Published:** 2025-06-10

**Authors:** Christoph Lehmann, Deoraj Singh, Maria Gastearena, Laura M. Comella

**Affiliations:** a Cluster of Excellence livMatS @ FIT – Freiburg Center for Interactive Materials and Bioinspired Technologies, University of Freiburg Freiburg Germany christoph.lehmann@livmats.uni-freiburg.de; b Laboratory for the Design of Microsystems, Department of Microsystems Engineering, University of Freiburg Freiburg Germany; c Laboratory for Chemistry and Physics of Interfaces, Department of Microsystems Engineering, University of Freiburg Freiburg Germany; d Institute of Energy Efficient Mobility, Karlsruhe University of Applied Sciences Karlsruhe Germany

## Abstract

Microfluidic devices, especially those utilizing polydimethylsiloxane (PDMS) structures, require reliable bonding methods to achieve durable, leak-proof seals. Current bonding techniques, including O_2_ plasma treatment, suffer from limitations related to material compatibility and surface roughness sensitivity, which compromise device stability and scalability in complex designs. In this study, we investigate the impact of surface roughness, wax contamination, and the presence of conductive materials on bonding strength in PDMS-based microfluidics. Additionally, we propose a novel bonding method using a flowable, one-component silicone rubber that forms robust seals without plasma treatment or silanization, effectively overcoming the challenges posed by increased surface roughness and material heterogeneity. The bonding method demonstrated significantly enhanced bond strengths across various substrate combinations (PDMS, copper, and FR4), with notable resilience under high pressure. This approach advances microfluidic fabrication by offering a scalable, versatile solution for multi-material bonding applicable in digital microfluidics and beyond.

## Abstract 2

A novel bonding method using silicone rubber provides robust bonding for silicones, metals, and epoxies without plasma treatment, overcoming surface roughness challenges for scalable multi-material microfluidic device fabrication.

## Graphical abstract

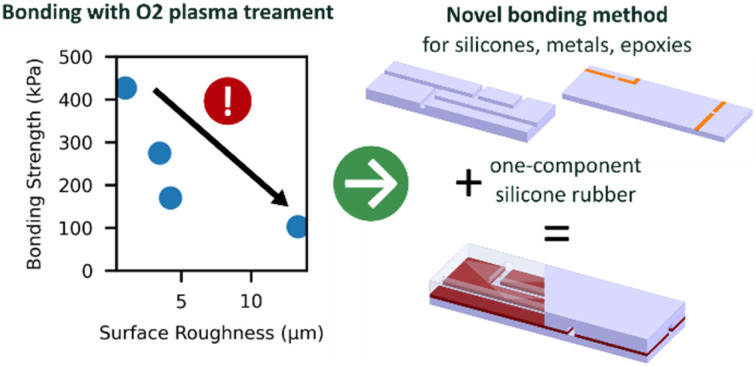

## 1 Introduction

Microfluidic devices offer various advantages in fields such as lab-on-a-chip testing in clinical diagnosis,^1^ separation and detection of analytes,^2^ laboratory automation technologies^3^ and on-site monitoring in environmental sensing.^4^ The physical dimensions of microfluidic devices offer advanced applications with a high degree of complexity on a small footprint. As a result, microfluidics have raised widespread interest across many disciplines.

While conventional microfluidics has proven valuable in the applications mentioned above, it faces certain limitations. The fixed nature of microchannels can restrict the degrees of freedom for fluid manipulation, and scaling up operations often requires complex redesigns.^5,6^ These challenges have led to the emergence of digital microfluidics. Unlike conventional microfluidics, digital microfluidics relies on an array of electrodes to manipulate discrete droplets or fluid phases on a planar surface.^7^ This electrode-based approach uses electrical fields to control droplet movement, merging, splitting, and mixing operations. Moreover, electrodes can be utilized to measure the electrical properties of the droplets, such as impedance^8^ or capacitance,^9^ enabling integrated sensing capabilities. This dual functionality of actuation and sensing makes digital microfluidics particularly attractive for lab-on-a-chip and lab-on-PCB applications, where fluid manipulation and analysis are required on a single platform.^10,11^

A microfluidic channel in both conventional and digital microfluidics can be implemented into a variety of materials, such as glass,^12^ silicones, and in particular polydimethylsiloxane (PDMS).^13^ Manufacturing microfluidic devices using PDMS offers easy fabrication, and enables features including optical transparency, biocompatibility, and versatile functionalization chemistries.^13^ Microfluidic devices that are fully PDMS-embedded offer additional advantages like being flexible and conformal to their environment, opening up applications in new areas in soft robotics and sensing in shape-changing environments.^14–17^ For fast prototyping and easy fabrication, lab-on-PCB devices offer the advantages of high integration, personalized design and easy mass production.^11^ However, integrating electrodes within digital microfluidic devices remains a significant challenge for the development of flexible sensing and manipulation capabilities. Conventional methods for embedding electrodes microfluidic devices often involve complex and time-consuming processes such as multi-step lithography, and large electrode sizes.^8,9,18^ Further, sometimes electrode materials with a high resistance are used, which can increase the error during signal acquisition.^18^

Regardless of the design and fabrication method, the fabricated open microchannels must be closed with another substrate to create a leakage-free device. This step in the fabrication process is especially critical in digital microfluidics since a tight and secure bond between various materials including silicone, metal, epoxy, and other polymers with a single bonding technique needs to be realized. As the microchannel dimensions approach low order micrometer scale and the network complexity increases, a bonding technique needs to be consistent over multiple length scales.^19^ To the best of our knowledge, a unified method for PDMS–PDMS, metal-PDMS, and epoxy-PDMS bonding has not been proposed yet. This limitation is significant because digital microfluidic devices often require the integration of these various materials to function effectively. Moreover, the incorporation of electrodes within flexible PDMS structures often necessitates trade-offs between mechanical flexibility and electrical functionality. Existing methods to address these challenges can involve labor-intensive and costly fabrication steps or require clean room facilities, limiting their scalability and accessibility for widespread.^20^

Conventional bonding methods in microfluidic device fabrication with PDMS typically involve oxygen plasma treatment, which temporarily renders the PDMS surface hydrophilic, allowing it to bond strongly with glass or another PDMS layer upon contact. However, the method does not work for bonding PDMS to metals, epoxies, or PMMA, having no silanol or hydroxyl groups to engage in a strong chemical bonding. Adhesion between the above-mentioned materials and PDMS can be improved by treating the surface with silane coupling agents (silanization), such as (3-aminopropyl)triethoxysilane (APTES) and (3-glycidyloxypropyl)trimethoxysilane (GPTMS), before the conformal contact to improve adhesion.^21–23^ Due to the strong reactivity of the silane coupling agents, the silane solution usually needs to be prepared immediately before the bonding process and cannot be used anymore after 30 to 60 min.^24^ Further, surfaces need to be free of any contamination to allow for a proper silanization of the surfaces.

Other bonding methods use a thin film of various kinds of adhesives that are applied by either spin-coating or a stamping method. After conformal contact, the adhesive film is cured. Agostini *et al.*^25^ used an ultraviolet (UV)-curable glue, whereas Li *et al.*^26^ explored the use of an epoxy-based adhesive. Here, the surfaces also needed to be plasma-treated or silanized before applying the adhesive thin film, followed by a curing step in a UV chamber or oven. Due to the chemical composition of the adhesive, the bonding can be compromised by certain basic fluids,^27^ leading to de-bonding depending on the application. Cao *et al.*^28^ proposed dimethyl-methylphenylmethoxy siloxane as an adhesive layer for bonding PDMS to glass. Chow *et al.*^29^ used a stamped uncured PDMS layer as an adhesive between PDMS and PMMA without any surface pretreatment, but could only achieve weak bonds.

Despite the high versatility of bonding techniques for digital microfluidics, several problems persist. Achieving uniform and reliable bonds over large areas remains challenging, especially as device designs become more complex and multiple material combinations need to be bonded together with one single technique.^30^ Furthermore, the proposed bonding methods do not give directions on the surface roughness and chemistry from further fabrication steps.^20,31^ Inconsistent bonding can lead to fluid leakage, reduced device performance, and failure.

These challenges are particularly pronounced in digital microfluidics, where the integration of different materials is crucial and the surface profile of all materials cannot be controlled jointly for all. For instance, additive manufacturing processes, such as 3D printing of molds for microfluidic devices, are likely to produce surfaces with significantly higher roughness than standard photolithography.^32^ Specifically, Fused Deposition Modeling (FDM) can yield surface roughnesses *S*_a_ > 10 μm. Similarly, lab-on-PCB integration^11,33^ often encounters inherent surface roughness due to the etching and lamination processes involved in PCB fabrication, where surface roughness can also reach tens of micrometers.

In this paper, we investigated how inhomogeneities and alterations in the fabrication process before the conformal bonding can affect the bonding strength of the interfacial layer that is needed to seal a microfluidic device. We investigated three steps in the fabrication process for PDMS-embedded digital microfluidics. As a reference for the integration of metal electrodes on PDMS, we used our recently proposed fabrication method for stretchable printed circuit boards based on PDMS and structured copper tracks.^34^ There, we identified three manufacturing steps and alterations, that deviated from conventional bonding methods and could affect the bonding strength: (1) the effect of previous wax contamination of the PDMS surface (2) a change in contact angle after O_2_ plasma treatment with conductive materials being present on the PDMS surface and (3) the effect of surface roughness of the surfaces to be bonded. We show, that previous wax contamination and the presence of conductive materials on the surface do not change the surface characteristics for a successful bonding using methods with O_2_ plasma treatment. On the other hand, bonding methods that involve O_2_ plasma treatment fail under increased surface roughness of the substrate. We produced substrates with varying surface roughness and quantified the effect of the surface roughness on the bonding strength using conventional O_2_ plasma treatment as the bonding method.

As a possible solution to this problem, we present a novel bonding method that addresses this critical issue by utilizing a flowable, one-component silicone rubber. The silicone can be applied as a thin film to the bonding area, cures at room temperature under the influence of atmospheric moisture, and forms a tight bond to various substrates. The bonding method does neither require O_2_ plasma treatment nor surface silanization and therefore is insensitive to deviations caused by the above-mentioned problems during the manufacturing process. We evaluated the bonding strength between PDMS and PDMS, copper and FR4 (fiberglass-epoxy composite material) with varying surface roughnesses and compared our results to the currently existing bonding methods.

## 2 Methods and materials

### 2.1 Surface preparation and characterization

**Fig. 1:**
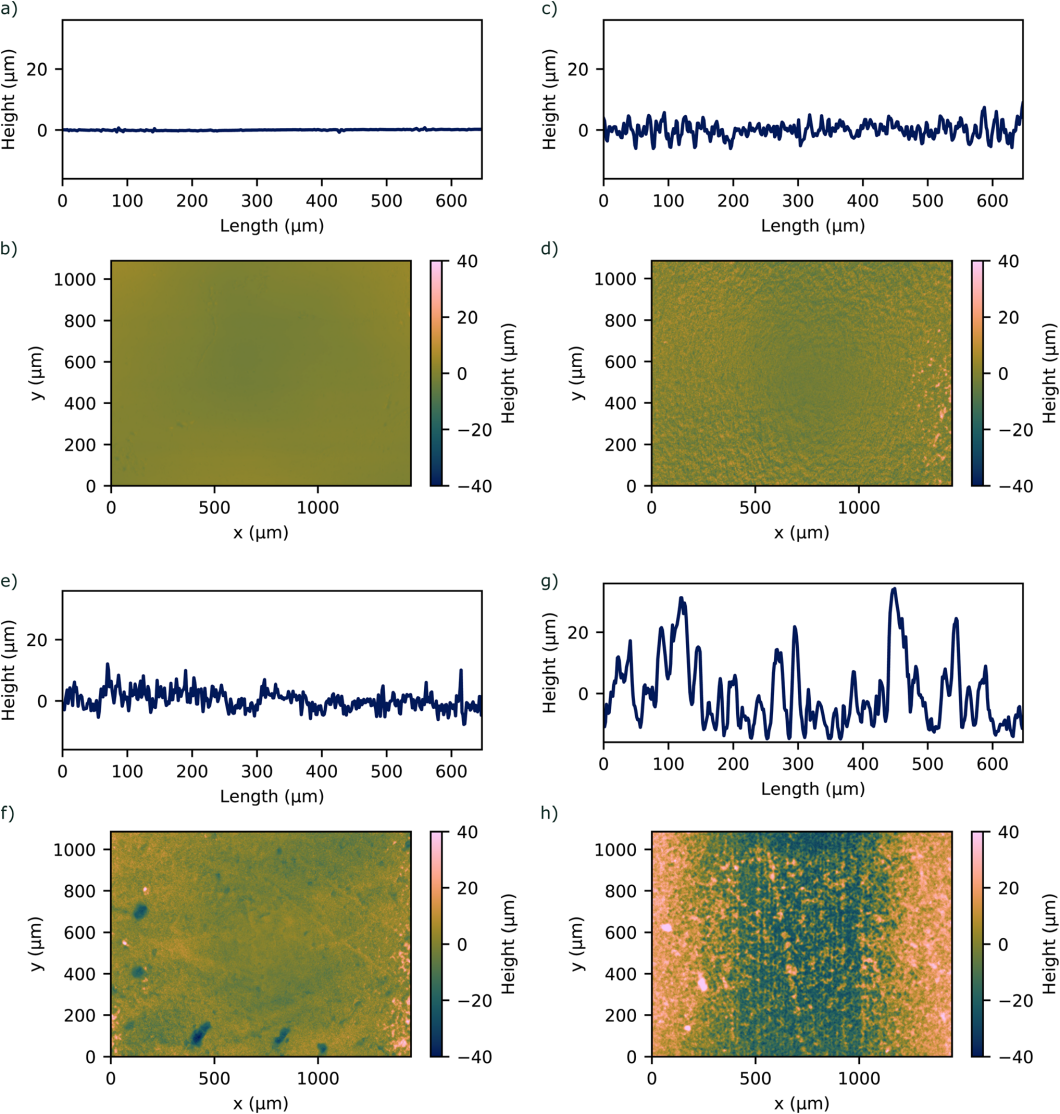
1D and 2D surface profile of investigated PDMS surfaces with corresponding surface roughness of (a and b) *S*_a_ = 1.00 μm (c and d) *S*_a_ = 4.24 μm (e and f) *S*_a_ = 3.47 μm (g and h) *S*_a_ = 13.34 μm.

To investigate the bonding strength under varying surface roughnesses, samples were prepared for tensile tests by using molds with open bottoms placed on various substrates to replicate surfaces with different surface roughnesses. The prepolymer and the curing agent (RT 601, Wacker, Germany) were mixed at a 9 : 1 ratio by weight, degassed to remove air bubbles, and poured into the molds placed on the respective substrate. The PDMS was cured at 70 °C for 1 h. After curing, the PDMS samples were carefully removed from the substrates, creating a set of samples with distinct surface roughnesses determined by the profile of the underlying substrate. The surface roughness was measured using a laser scanning microscope (VK-X3000, Keyence, Japan) to quantify the topography of the PDMS samples. The roughness profile was determined by scanning across a representative area for each sample. Fig. 1 shows representative line roughness profiles and surface profiles on the bonding area of the PDMS samples. We achieved surface roughnesses *S*_a_ in the range between 1.00 μm and 13.34 μm.

To characterize possible contamination during fabrication processes, pristine PDMS samples were prepared as the base material. To simulate wax contamination, a wax layer (EM-Tec TempStick 135C, Micro to Nano, Netherlands) was melted onto the surface of select PDMS samples and left to fully cool. The wax-contaminated PDMS samples were then cleaned using acetone. Three sample types were prepared for Fourier transform infrared spectroscopy (FTIR) analysis: (i) pristine PDMS, (ii) waxed PDMS, and (iii) waxed PDMS cleaned with acetone. The FTIR measurements were performed using a spectrometer (Cary 630 FTIR, Agilent, USA) in transmission mode. Spectra were collected in the wavenumber range of 650 cm^−1^ to 4000 cm^−1^ to analyze the chemical composition of the surface materials. For each sample, transmittance data was recorded as a function of the wavenumber.

### 2.2 Contact angle measurement

The contact angle of four distinct surfaces was evaluated: PDMS with a surface roughness of *S*_a_ = 1.00 μm and *S*_a_ = 4.24 μm, PDMS (*S*_a_ = 4.24 μm) with in-plane copper electrode and copper (*S*_a_ = 3.47 μm, *S*_a_ = 13.34 μm). All samples were characterized by contact angle measurements using a goniometer (OCA 15 EC, DataPhysics Instruments, Germany) before and after O_2_ plasma treatment with at least three readings each.

### 2.3 Bonding method

**Fig. 2:**
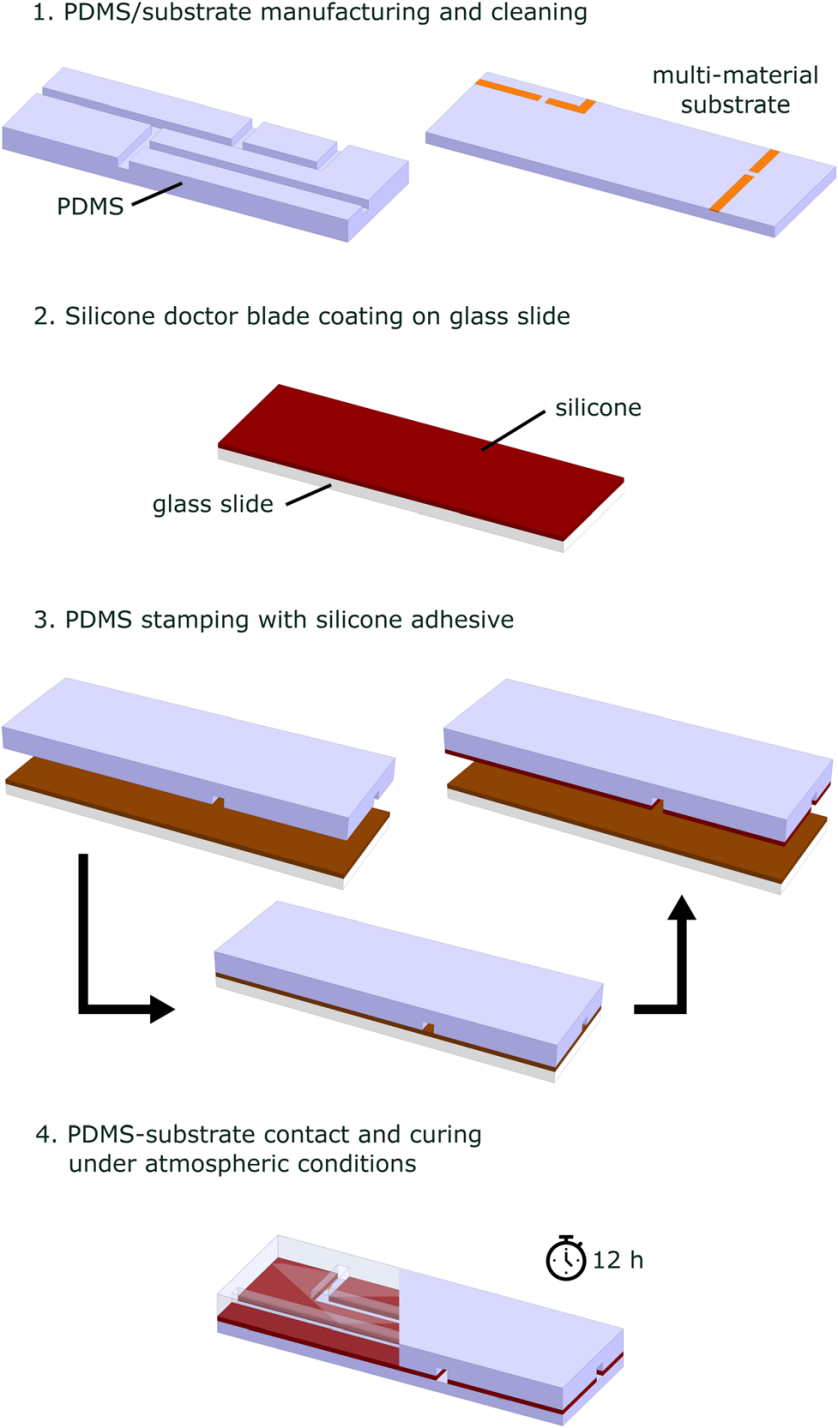
Bonding process using a one-component silicone rubber as sealant.

Prior to the bonding process, the substrate and microchannel matrix were fabricated. For a PDMS substrate with integrated co-planar electrodes and electrical pathways, our previously reported method for stretchable and flexible PCBs was used.^35^ In this work, the microchannel was made by 3D-printing a master mold of the structure on a glass slide using a high-resolution SLA printer (Dilase 3D, Kloé, France). PDMS was subsequently cast over the master mold to form the negative of the 3D-print into the PDMS matrix. The bonding process between PDMS and a multi-material substrate, shown in Fig. 2, starts by cleaning both surfaces with acetone and drying under an air stream. The one-component silicone rubber (A07, Wacker, Germany) was doctor-blade coated onto a glass slide to create a thin film with a thickness of 100 μm. The film was then transferred to the PDMS surface by putting in contact the two surfaces for 5 s. Subsequently, the PDMS surface with the silicone rubber and the substrate were put into contact and cured for minimum 12 h under atmospheric conditions. Since PDMS is gas permeable, moisture could penetrate the structure and fully cure the silicone rubber thin film.

### 2.4 Bonding strength measurement

The bonding strength between two substrates was quantified by performing a cylinder-based tensile strength measurement^36^ with specially designed PDMS pieces, see Fig. S1 ESI.† To investigate the effect of the surface roughness on the bonding strength between two PDMS substrates, we molded PDMS specimens that had varying surface roughness on the surface to be bonded, see Fig. 1. For the bonding strength test using O_2_ plasma treatment, the specimens were treated under an O_2_ plasma (Zepto One, Diener, Germany) for 1.75 min and then brought into conformal contact with each other. Between the two surfaces, one surface roughness was kept constant at *S*_a_ = 1.00 μm, while the other was changed.

To investigate the bonding strength of the one-component silicone rubber, we applied a thin film of the silicone rubber on the surface of the PDMS specimen, as described in the preceding section and then brought it into contact with the second substrate. We tested the bonding strength of the material combinations PDMS–PDMS, PDMS–FR4, and PDMS–copper. Between the two substrates, the PDMS surface roughness was kept constant at *S*_a_ = 1.00 μm, whereas the roughness for respective PDMS, FR4, and copper surface was changed. For FR4 and copper, we used the metal and non-metal sides of pristine copper-clad laminate (35/00 Cu, Bungard, Germany). We achieved different surface roughnesses by sanding the surface with sandpaper, the pristine surface profile of FR4 and copper is shown in Fig. S2 (ESI).† The bonded substrates were clamped into the tensile test machine (Inspect Table, Hegewald & Peschke, Germany), see Fig. S3 (ESI).† For all tensile tests, the test speed was set to 10 mm s^−1^. Force and displacement were simultaneously recorded. The bonding strength was calculated from the maximum force and the cross-section of the bonded interface. The bonding strength values reported are the average of at least three measurements.

## 3 Results and discussion

### 3.1 Surface characterization

**Fig. 3:**
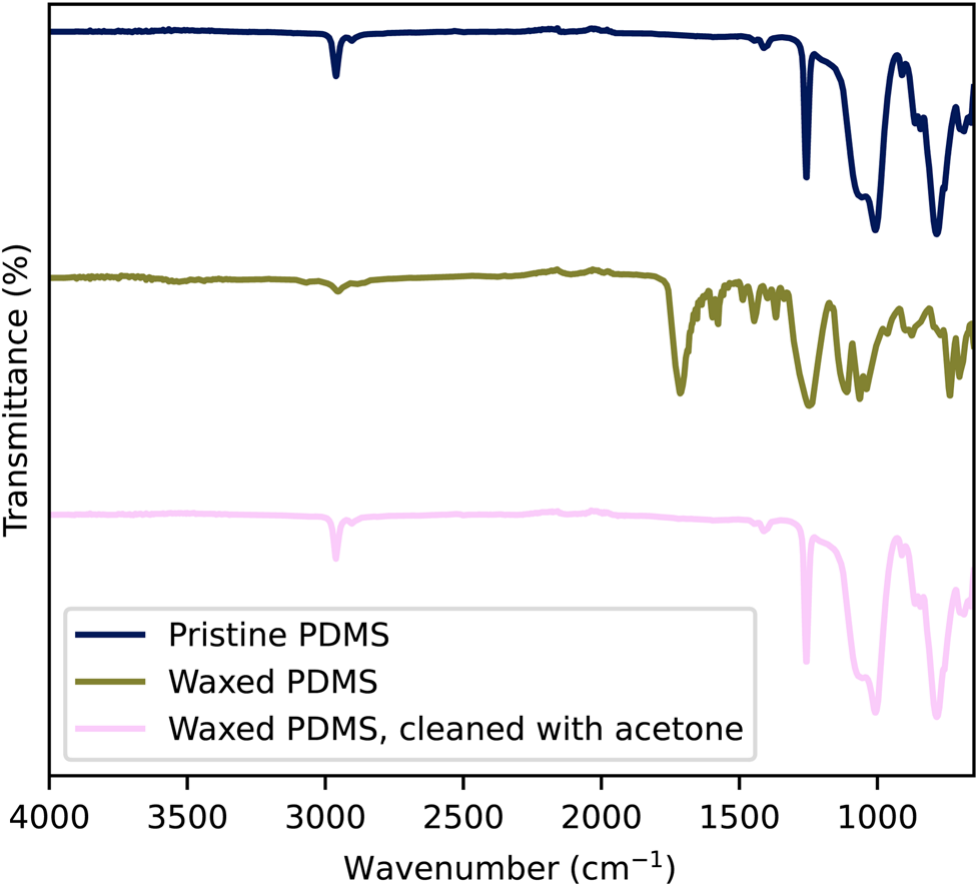
FTIR measurement of pristine PDMS, waxed PDMS cleaned with acetone and pristine wax.

Fig. 3 shows the FTIR spectra of pristine PDMS, wax-contaminated PDMS, and PDMS cleaned with acetone. Pristine PDMS exhibits characteristic peaks associated with PDMS, namely the symmetric bending of the Si–CH_3_ bond at 1255 cm^−1^ and the stretching of Si– O– Si bonds at 1000 cm^−1^. The wax layer, composed of a mixture of phthalic anhydride and ethylene glycol, shows a prominent peak at 1715 cm^−1^, corresponding to the C=O stretching of the anhydride group. Said stretching typically appears as two peaks, the asymmetric and symmetric stretching of the bond. The peaks may lack distinction because it is mixed with ethylene glycol. The C– O– C stretching is also visible at 1240 cm^−1^. The presence of ethylene glycol is evident by the broad band at 3400 cm^−1^, characteristic of O– H stretching, and the stretching of the C–O bond at 1060 cm^−1^. The lack of characteristic peaks associated with C– O bonds in the cleaned PDMS indicates that the acetone cleaning procedure effectively removed the wax contamination. We therefore concluded that the waxed PDMS surface, cleaned with acetone, can be considered equivalent to that of pristine PDMS. The surface is free of any contaminants and can be therefore used for further surface modifications such as the described silanization.

### 3.2 Contact angle measurement

The contact angle measurements before and after O_2_ plasma treatment (PT) are summarized in Table 1, representative contact angle recordings for each specimen are shown in Fig. S4 (ESI).† The contact angle readings before treating the pristine PDMS with O_2_ plasma show similar results as already reported.^37^ As described by Wenzel,^38^ the surface roughness exaggerates the wetting behavior. For the hydrophobic surfaces analyzed, the increasing roughness makes the contact angle larger and the surface appear more hydrophobic. Upon O_2_ plasma treatment, the contact angle for all samples decreased significantly, rendering the surfaces hydrophilic. Notably, the surface of specimen 3 could be rendered hydrophilic despite having a conductive copper electrode on the surface. Further, the surfaces could be rendered hydrophilic independent of the surface roughness of the substrate, indicating that further surface modifications like silanization can be performed. This is crucial, as many of the formerly mentioned bonding methods rely upon this step.

**Table 1:** Contact angle measurements for different materials and surface properties before and after O_2_ plasma treatment (PT)

Specimen	Surface	Contact angle (°)	
		Before PT	After PT
1	PDMS *S*_a_ = 1.0 μm	112 ± 3	12 ± 3
2	PDMS *S*_a_ = 4.24 μm	128 ± 3	9 ± 2
3	PDMS *S*_a_ = 4.24 μm with copper electrode	125 ± 2	27 ± 9
4	Copper *S*_a_ = 3.47 μm	110 ± 3	27 ± 6
5	Copper *S*_a_ = 13.34 μm	143 ± 3	22 ± 4

### 3.3 Bonding strength

#### 3.3.1 O_2_ plasma treatment

**Fig. 4:**
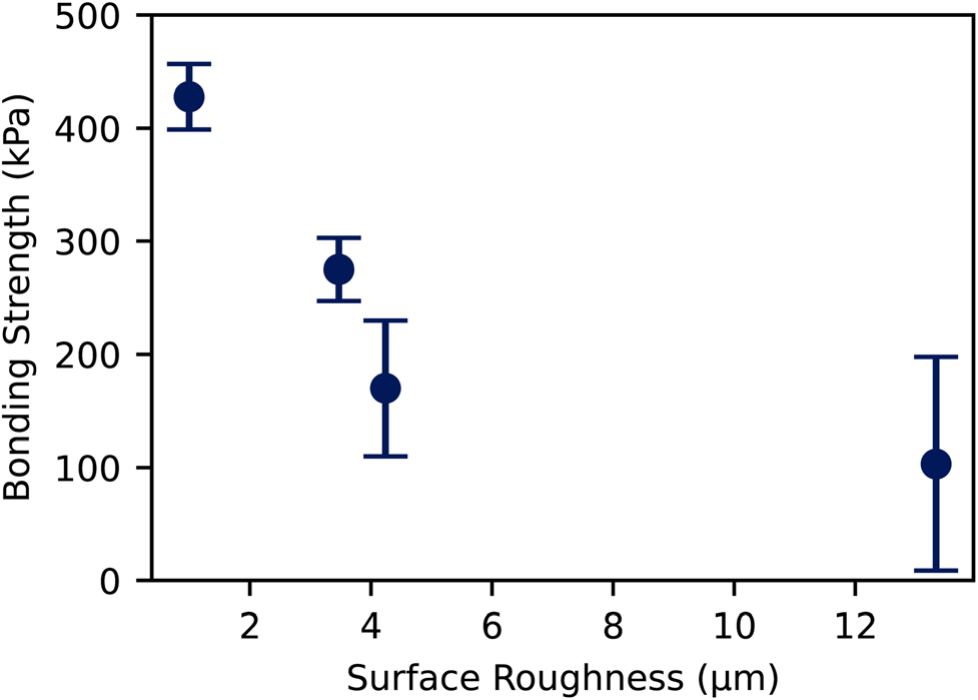
Tensile strength of two PDMS surfaces bonded by O_2_ plasma treatment. Surface 1 has a surface roughness *S*_a_ = 1.00 μm, the surface roughness of surface 2 is varied. The error shows the 68% confidence on mean.

The bonding strength of the substrates bonded using O_2_ plasma treatment showed a significant dependency on the surface roughness of the substrates, see Fig. 4. The maximum bonding strength was achieved at (428 ± 29) kPa with both surfaces having a roughness of *S*_a_ = 1.0 μm. As the surface roughness increased, we observed a strong decrease in bonding strength. With a surface roughness of *S*_a_ = 4.24 μm, the bonding strength decreased to 40% of the maximum value at (170 ± 60) kPa. At *S*_a_ = 13.34 μm, the bonding strength dropped to a degree that some specimens did not bond at all. The bonding strength dropped to (103 ± 95) kPa, 24% of the maximum observed value. Further, it can be seen that the bonding becomes less consistent, resulting in a higher standard deviation of the bonding strength with increasing surface roughness. The increased surface roughness results in fewer contact points between the two substrates. As the bonding strength scales with the effective bonding area, the decrease in bonding strength can be attributed to the reduction in the effective contact area between the substrates as the surface roughness increases. With higher roughness, the peaks and valleys on the surface prevent full contact over the entire area, limiting the formation of strong bonds. Additionally, surface irregularities can trap air or contaminants, further diminishing the adhesion quality. This explains the greater variability in bonding strength observed at higher roughness levels, as the number of effective bonding sites becomes more inconsistent. Therefore, controlling the surface roughness is crucial for optimizing bonding strength in O_2_ plasma-treated substrates.

#### 3.3.2 One-component silicone rubber

**Fig. 5:**
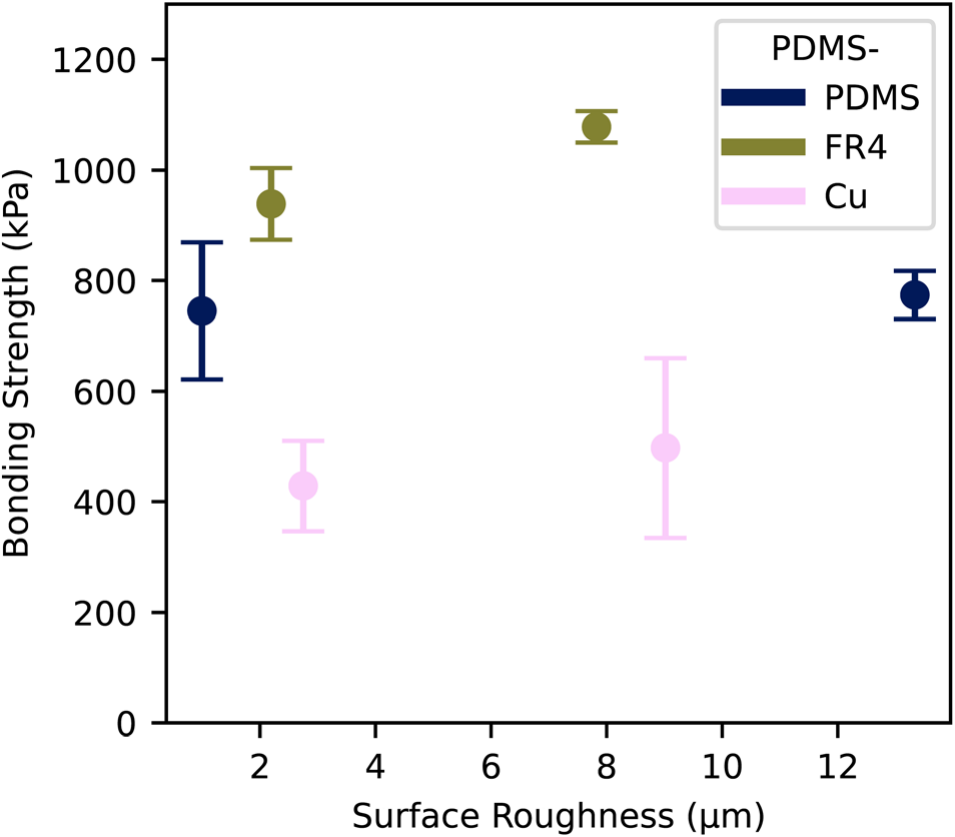
Tensile strength of substrates bonded using an interfacial layer of one-component silicone rubber, with surface roughnesses *R*_a_PDMS__ = 2.44 μm. The error shows the 68% confidence on mean.

The bonding strength for the three substrates using a one-component silicone rubber is presented in Fig. 5. All bonded material combinations exhibited a high bond strength over 400 kPa, making it ideal for high-pressure microfluidic applications. Conversely, we did not observe a decrease in bonding strength with an increase in surface roughness, but rather a slight increase for all substrate types in the range between 3.7% and 16.1%. We assume, that the increased surface roughness leads to an increased effective surface area. As the silicone rubber is flowable, the higher effective surface area resulted in a higher bonding area and thus higher bonding strength. We expect similar effects for other bonding methods with an intermediate adhesive layer.^25,26,39^

The maximum achieved bonding strength with our proposed method is summarized together with other previously suggested bonding methods in Table 2. FR4 exhibited the highest bonding strength with PDMS with a maximum of (1078 ± 28) kPa, 183% more than the highest reported value.^39^ The maximum PDMS–PDMS bonding strength was (773 ± 123) kPa, 18% higher than the highest reported value. Similarly, the maximum bonding strength for PDMS and copper was achieved at (498 ± 162) kPa, 59% higher than the highest reported value. In addition to the higher bonding strength, our proposed method does not need any surface functionalization using corona or O_2_ plasma treatment and bonds well without any surface silanization. The curing time for our method, at 12 hours, is longer compared to the methods reported previously. In addition, as the silicone rubber film is thin, it cures within 2 min under atmospheric conditions. The curing time could be extended or stopped by reducing the humidity or working within inert conditions.

**Table 2:** Comparison of achieved bonding strengths for various methods to bond PDMS with PDMS, FR4 and metals (RT = room temperature)

Reference	Bonded substrates	Bonding strength	Bonding method (kPa)	Temperature	Time
Our work	PDMS–PDMS (*S*_a_ = 1.00 μm/13.34 μm)	773 ± 123	One-component silicone rubber	RT	12 h
	PDMS–FR4 (*S*_a_ = 1.00 μm/7.83 μm)	1078 ± 28			
	PDMS–Cu (*S*_a_ = 1.00 μm/9.02 μm)	498 ± 162			
Wu *et al.*^24^	PDMS–Cu	236	Corona discharge treatment followed by surface modification with 2% MPTMS	RT	≤1 h
Sunkara *et al.*^21^	PDMS–Cu	312 ± 57	O_2_ plasma treatment followed by surface modification with 1% APTES	RT	≤1 h
Chang and Yu^39^	PDMS–PDMS	650	Half-cured PDMS film	65 °C	30 min
	PDMS–FR4	380			
Lee and Chung^23^	PDMS–PDMS	184	O_2_ plasma treatment followed by surface modification with 1% APTES and GPTMS	RT	1 h
Vlachopoulou *et al.*^40^	PDMS–PDMS	406	O_2_ plasma treatment followed by surface modification with 5% APTES	RT	1 h
Samel *et al.*^41^	PDMS–PDMS	545	Spin coating of PDMS curing agent	65 °C	4 h
Agostini *et al.*^25^	PDMS–PDMS	(2 bar)	O_2_ plasma treatment followed by surface modification with 1% APTES + spin coating of UV-curable glue	RT	≤1 h

### 3.4 Application

**Fig. 6:**
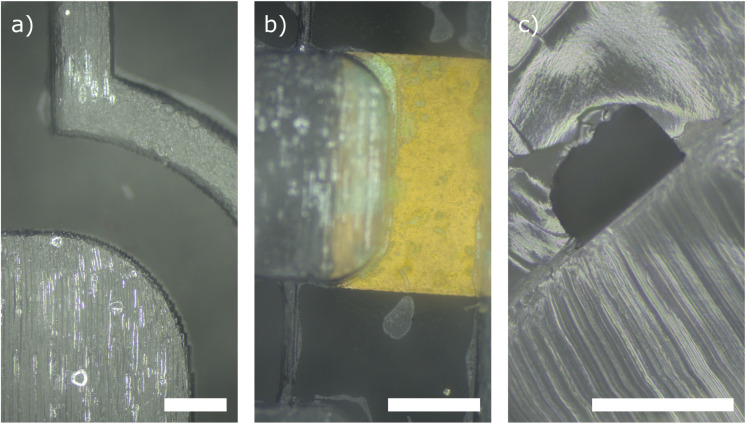
Microscope image silicone rubber bonding with (a) top view of microchannels (b) PDMS–PDMS and PDMS–copper bonding and (c) cross section of microchannel with dimensions 500 μm × 350 μm. The scale bar corresponds to 500 μm.

To showcase the performance and usability of our proposed fabrication method, we have manufactured fully PDMS-embedded microchannels with integrated electrodes. Fig. 6(a and b) shows two top view microscope images of microchannels fully embedded into a transparent PDMS matrix. The channel was closed using a one-component silicone rubber. Fig. 6(b) shows the bonding of the microchannel matrix with a multi-material substrate made of copper and PDMS. We achieved a reliable, leak-free bond. Further, we were able to bond the microfluidic device without clogging the channels with silicone rubber. The minimum channel width analyzed was 270 μm. Fig. 6(c) shows a cross-sectional view of a microchannel with a cross section of 500 μm × 350 μm. The image does not show any clogging or spilling of the adhesive layer into the channel, indicating that even smaller microchannel dimensions can be realized with our method.

## 4 Conclusions

In this paper, we studied three possible reasons for the lack of bonding strength in the field of microfluidics: contamination, O_2_ plasma treatment failure, and surface roughness. We showed that prior wax contamination and conductive materials do not alter the surface modification for manufacturing processes like O_2_ plasma treatment and surface silanization. On the other hand, we could show that the surface roughnesses of the surfaces to be bonded have a significant influence on the final bonding strength when O_2_ plasma treatment is used. We assume, that this effect derives from the decreasing effective bonding area as more pronounced peaks and valleys on the surface occur with increased surface roughness. As shown, many proposed bonding methods rely on a combination of O_2_ plasma treatment and surface silanization. There, the surface roughness is defined by the substrate's surface profile. As the surface roughness does not change on a macroscopic level during the surface modification, we believe that the bonding strength dependency on the surface roughness persists with these bonding methods as well and should be considered in fabrication processes and studies developing bonding technologies. This hypothesis will be investigated in a future study.

Our newly proposed method uses a one-component silicone rubber which is applied as a thin film on the substrate and acts as a glue between the two substrates upon conformal contact. Since the used silicone rubber is flowable, it can compensate for increased surface roughnesses and inhomogeneities on the substrate surface, making it a useful bonding agent for various types of surfaces. We could show that the bonding method works between PDMS and PDMS, copper and FR4, making it ideal especially in the field of digital microfluidics. Further, it does not require prior O_2_ plasma treatment and surface modification. To the best of our knowledge, this is the first reported bonding method, that does not require prior plasma treatment and surface silanization and still achieved bonding strengths in the same order as previous reported methods.

## Author contributions

Christoph Lehmann: Conceptualization, methodology, investigation, formal analysis, writing – original draft Deoraj Singh: Investigation, data curation Maria Gastearena: Investigation, writing – reviewing and editing Laura M. Comella: Funding acquisition, supervision, writing – reviewing and editing.

## Conflicts of interest

There are no conflicts to declare.

## Supplementary Material

RA-015-D5RA02701B-s001

## Data Availability

The data supporting the findings of this study are included within the manuscript and its ESI.† The raw data associated with this manuscript is available from the corresponding author upon request.
