# Supplementary material for: Surface roughness in microfluidic device fabrication: limitations of conventional methods and a novel solution for multi-material bonding
Source: RSC Adv. 2025 Jun 10;15(24):19254–62. doi: 10.1039/d5ra02701b (PMC12150281; doi:10.1039/d5ra02701b)
Supplement: RA-015-D5RA02701B-s001 [file RA-015-D5RA02701B-s001.pdf]

Supplementary Information (SI) for RSC Advances.
This journal is © The Royal Society of Chemistry 2025

Supporting Information for

Surface roughness in microfluidic device fabrication: Limitations of conventional methods and a novel solution for multi-material bonding

Christoph Lehmann[a,b], *Deoraj Singh*[a], *Maria Gastearena*[a,c], *Laura M. Comella*[a,d]

[a] Cluster of Excellence livMatS @ FIT – Freiburg Center for Interactive Materials and Bioinspired Technologies, University of Freiburg, Freiburg, Germany
[b] Laboratory for the Design of Microsystems, Department of Microsystems Engineering, University of Freiburg, Freiburg, Germany
[c] Laboratory for Chemistry and Physics of Interfaces, Department of Microsystems Engineering, University of Freiburg, Freiburg, Germany
[d] Faculty of Engineering and Mechatronics, Karlsruhe University of Applied Sciences, Karlsruhe, Germany

Email: christoph.lehmann@livmats.uni-freiburg.de

Fig. S1.

Geometry of the PDMS specimen with **a)** CAD rendering of PDMS specimen **b)** specimen dimensions. The specimens were casted in a 3D-printed mold (Form 3, Formlabs, USA) shaped as the negative. The mold had an open bottom to realize different surface roughness for the tensile test

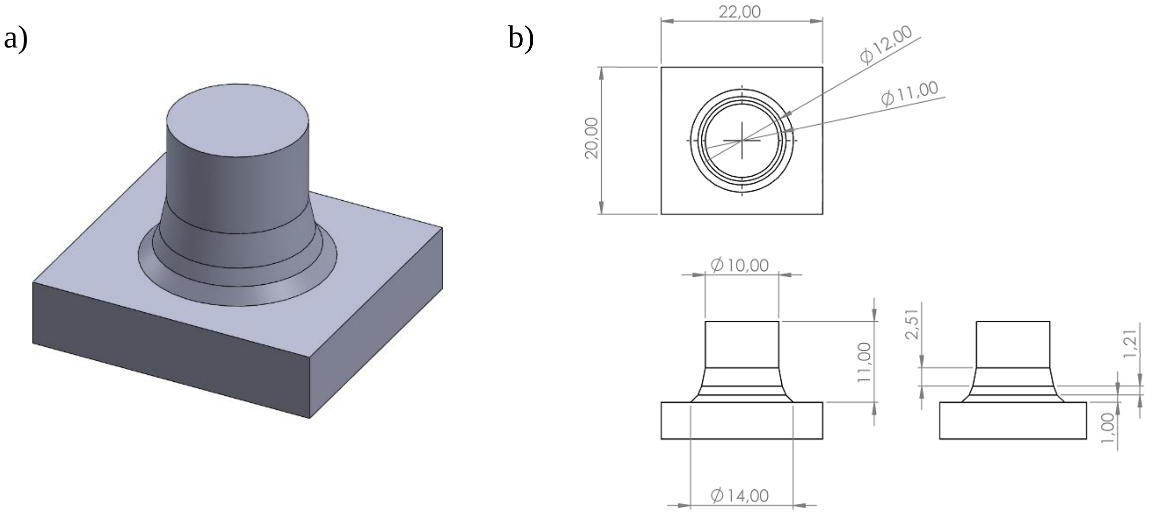

Fig. S2.

Surface profile of a) pristine FR4 and b) copper side of copper-clad laminate (Cu 35/00, Bungard, Germany)

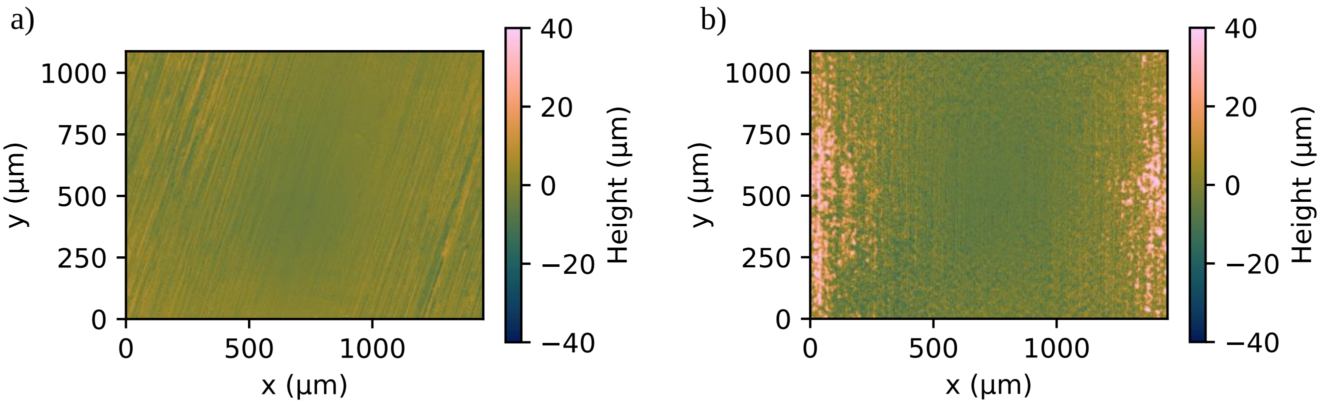

Fig. S3.

Tensile test setup with **a)** front view of tensile test machine with clamped specimen **b)** close-up of specimen clamped between the crossheads.

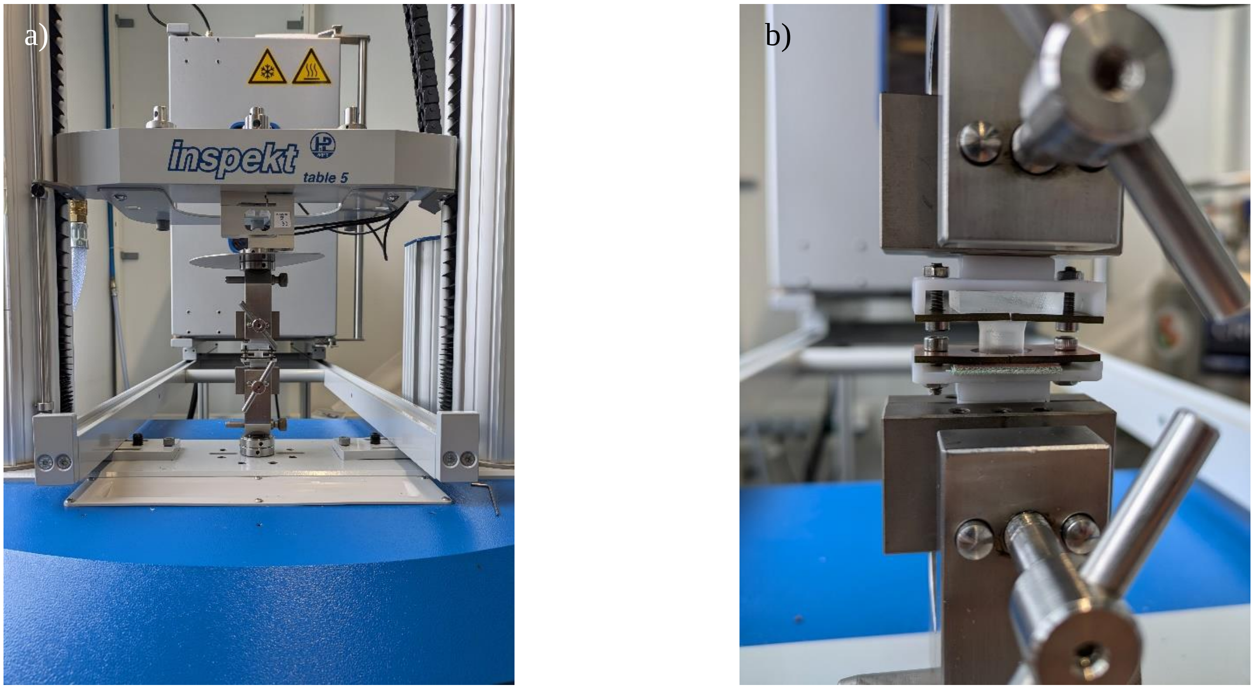

Fig. S4.

Representative contact angle measurement readings **a,c,e,g,i)** before and **b,d,f,h,j)** after O2 plasma treatment for **a,b)** PDMS (S_a = 1.0 µm) **c-d)** PDMS (S_a = 4.24 µm) **e,f)** PDMS (S_a = 4.24 µm) with present copper electrode **g,h)** copper (S_a = 3.47 µm) **i,j)** copper (S_a = 13.34 µm)

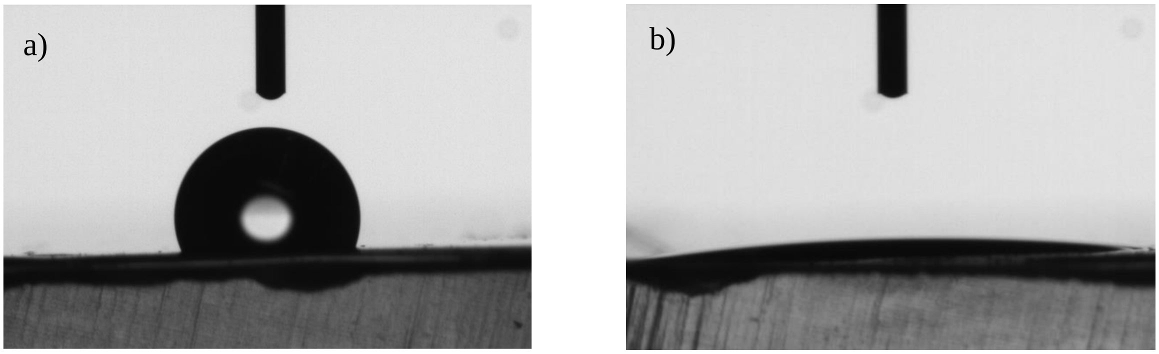

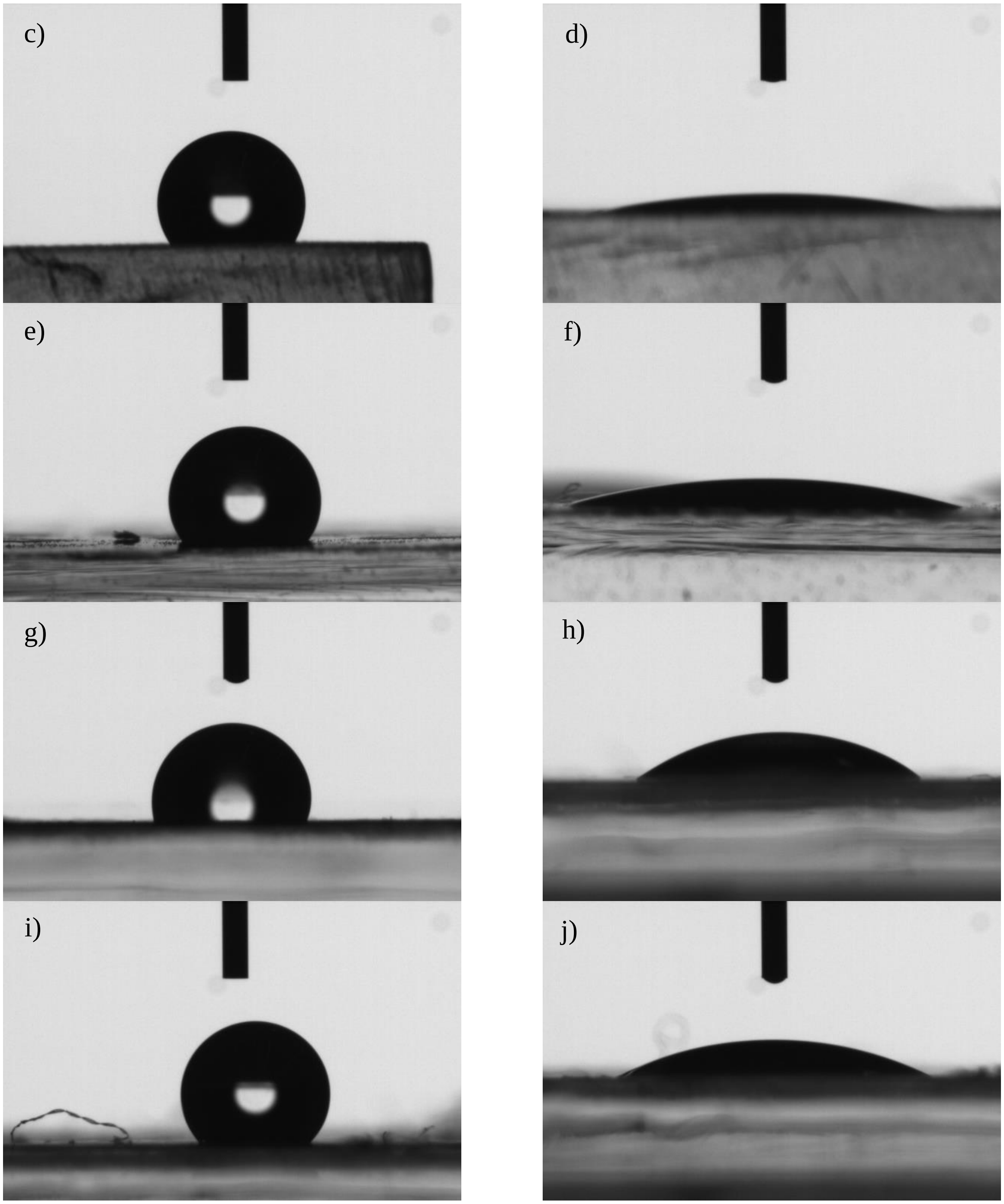
c)
d)
e)
f)
g)
h)
i)
j)
